# Tuning Antioxidant Function through Dynamic Design of Chitosan-Based Hydrogels

**DOI:** 10.3390/gels10100655

**Published:** 2024-10-13

**Authors:** Manuela Maria Iftime, Gabriela Liliana Ailiesei, Daniela Ailincai

**Affiliations:** 1 “Petru Poni” Institute of Macromolecular Chemistry, Grigore Ghica Voda Alley, 700487 Iasi, Romania; gdarvaru@icmpp.ro (G.L.A.); ailincai.daniela@icmpp.ro (D.A.); 2 The Research Institute of the University of Bucharest (ICUB), 90 Sos. Panduri, 050663 Bucharest, Romania

**Keywords:** chitosan, imine, hydrogels, dynamic behavior, antioxidant properties

## Abstract

Dynamic chitosan-based hydrogels with enhanced antioxidant activity were synthesized through the formation of reversible imine linkages with 5-methoxy-salicylaldehyde. These hydrogels exhibited a porous structure and swelling capacity, influenced by the crosslinking degree, as confirmed by SEM and POM analysis. The dynamic nature of the imine bonds was characterized through NMR, swelling studies in various media, and aldehyde release measurements. The hydrogels demonstrated significantly improved antioxidant activity compared to unmodified chitosan, as evaluated by the DPPH method. This research highlights the potential of developing pH–responsive chitosan-based hydrogels for a wide range of biomedical applications.

## 1. Introduction

Oxidative stress is a critical factor in the pathogenesis of various diseases, arising from an imbalance between reactive oxygen species (ROS) and the body’s antioxidant defenses [1]. It contributes to various diseases, including cancer, heart disease, neurodegenerative disorders, and chronic inflammation. Maintaining a balance between free radicals and antioxidants is crucial for overall health. To address this, adaptable antioxidant delivery systems are essential for enhancing antioxidant efficacy and targeting specific diseases. These systems are designed to improve the pharmacological and therapeutic properties of antioxidants by enhancing their stability, bioavailability, and targeted delivery. Some of the most commonly used adaptable antioxidant delivery systems include nanoparticles [2], liposomes, nanoemulsions, cyclodextrins, polymeric micelles [3], and hydrogels [4].

Hydrogels have garnered significant attention for antioxidant delivery due to their versatile properties, including biocompatibility, tunability, and controlled release capabilities [5,6]. In recent years, researchers have successfully tailored hydrogel systems, whether natural or synthetic, chemically or physically crosslinked, to specific antioxidant delivery applications [7,8].

Among these, chitosan-based hydrogels have developed as promising candidates due to their inherent biocompatibility, biodegradability, antimicrobial, and anticancer properties [9]. The ability to modify chitosan through various chemical reactions enables the creation of hydrogels with tailored properties, including enhanced antioxidant capacity [10,11]. Chitosan-based hydrogels can be prepared through physical methods such as hydrogen bonding, freeze-thawing, and ionotropic gelation [12] or chemical approaches including grafting, free-radical polymerization, enzymatic reactions, and condensation reactions [13,14].

It is well-established that dynamic covalent bonds are crucial for developing responsive and adaptable materials [15]. Among the various dynamic covalent bonds employed, including disulfide, hydrazone, oxime, boronate ester, and thioester bonds [16], imine linkages have gained importance for designing materials with specific antioxidant properties [17].

By incorporating dynamically reversible imine linkages, chitosan hydrogels gain enhanced functionality, enabling responsiveness to microenvironmental changes and modulated antioxidant release [18,19]. The pH sensitivity of these imine-linked chitosan hydrogels is particularly promising for biomedical applications [20,21]. The acidic microenvironments of tumors or wound sites can trigger imine bond hydrolysis and reformation, facilitating controlled drug release and tissue regeneration [22]. While dialdehydes have been commonly used as chitosan hydrogel crosslinkers, their potential cytotoxicity limits their applicability in biomedical contexts.

To address this, previous studies have focused on utilizing natural monoaldehydes with various properties, including antimicrobial [23], anticancer [24], and antioxidant activities [23,25,26,27]. These chitosan-based hydrogels exhibit excellent physicochemical and biological properties, including porous morphology [28], self-healing [29], swelling capabilities, and antibacterial activity [30]. These properties make them suitable candidates for various biomedical applications, including drug delivery and wound dressings [23,30].

Chitosan modification with 5-methoxysalicylaldehyde or its derivatives has been explored in several studies [31,32,33]. These studies focused on the synthesis of chitosan-Schiff bases and their potential applications, including antimicrobial and antitumoral activities, investigating the impact of various substituents on their properties or the formation of metal complexes with their derivatives [31].

Starting from previous results, the present study aims to design and characterize novel pH–responsive chitosan-based hydrogels based on reversible imine linkages. The use of 5-methoxy-salicylaldehyde, a compound known for its biocompatibility, antimicrobial activity, and antioxidant properties, further enhances the hydrogel’s functionalities for biomedical applications. Additionally, the dynamic nature of the imine linkages, formed between chitosan and the aldehyde, is expected to significantly influence the antioxidant properties of these hydrogels, allowing them to respond to environmental changes.

By investigating the structure-property relationships and dynamic behavior of these hydrogels, we also aim to elucidate their potential as adaptive materials for biomedical applications. A comprehensive characterization, including Nuclear Magnetic Resonance spectroscopy (NMR), Fourier–Transform Infrared Spectroscopy (FTIR), Scanning Electron Microscopy (SEM), and Polarized Optical Microscopy (POM) analysis, was conducted to understand the impact of imine bond formation on hydrogel properties. Additionally, the pH–dependent release of aldehyde and the antioxidant activity of the hydrogels were evaluated.

## 2. Results and Discussion

**Scheme 1:**
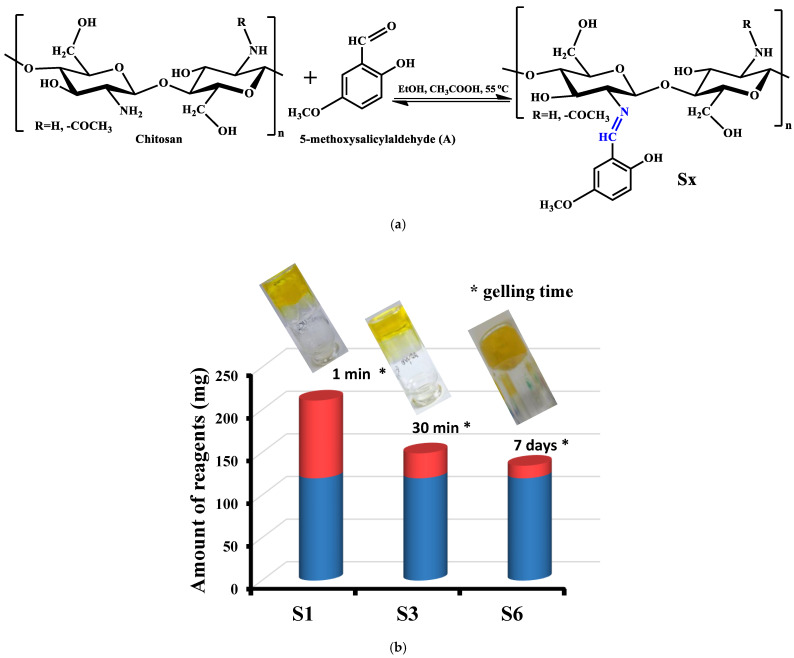
(**a**) Chitosan imination reaction with 5-methoxysalicylaldehyde; (**b**) Overview of hydrogel composition, gelation time, and inverted tube test images.

**Scheme 2:**
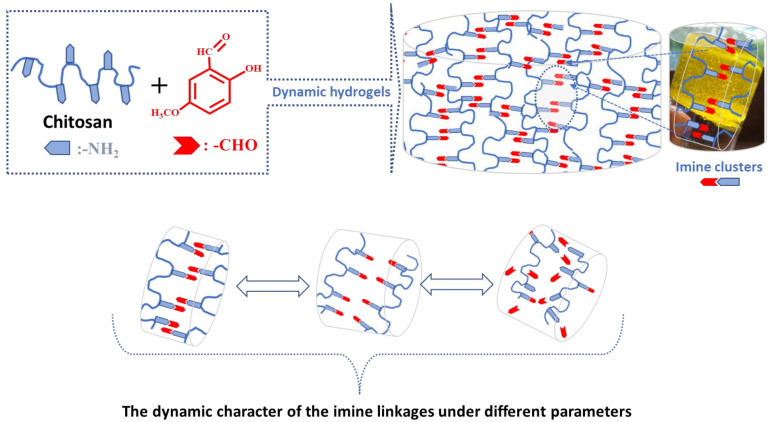
Schematic representation of hydrogel formation (**Sx**) and their dynamic properties attributed to reversible imine units.

Three hydrogels with various crosslinking degrees were synthesized through the acid condensation reaction of chitosan with 5-methoxysalicylaldehyde (**A**). The chitosan amount was maintained constant, while the aldehyde quantity was adjusted to achieve molar ratios of glucosamine units to aldehyde groups of 1/1, 3/1, and 6/1 (Scheme 1a,b). The schematic representation of their obtaining is shown in Scheme 2. Hydrogelation was a consequence of the formation of imine units on chitosan, which, due to the hydrophobic/hydrophobic aggregation, form clusters that act as physical crosslinking nodes for the chitosan chains (Scheme 1a) [24,25].

The reversible character of the imine units is expected to endow the hydrogels with a dynamic character, responding under the influence of external stimuli, such as pH or aldehyde consumption in various processes, e.g., the binding of free radicals. To prove this assumption, the formation of the hydrogels was monitored by structural and supramolecular investigations by ^1^H-NMR, FTIR, SEM, and POM analysis, and their dynamic character was investigated by ^1^H-NMR spectroscopy, aldehyde release under different pH conditions, and antioxidant activity measurements.

### 2.1. Structural Characterization

**Figure 1:**
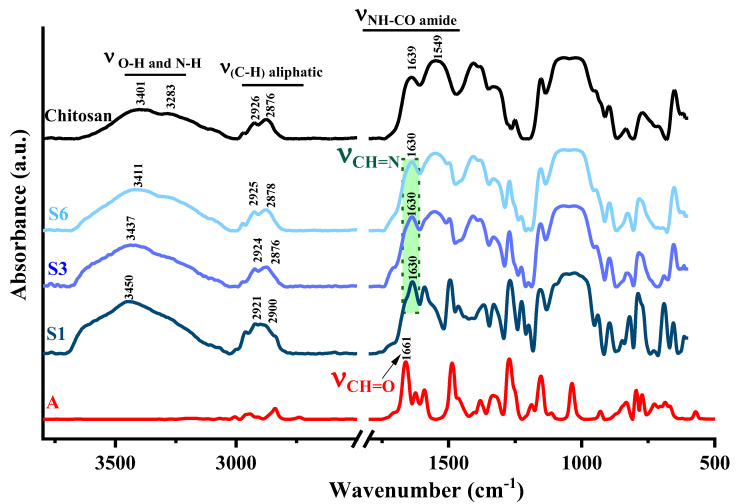
The FTIR spectra of the xerogels (**S1**, **S3** and **S6**), chitosan, and 5-methoxysalicylaldehyde (**A**).

First, the chemical reaction supporting the hydrogelation was verified by FTIR spectroscopy. The comparison of the hydrogel FTIR spectra with those of neat chitosan and aldehyde precursors showed clear modifications in line with the formation of the imine bonds (Figure 1). Thus, in the fingerprint region, a new sharp and intense band appeared at 1630 cm^−1^, attributed to the new imine bonds formed by aldehyde’s grafting to the chitosan chains [34,35,36]. The band at 1639 cm^−1^, characteristic of the amide bonds of chitosan was, still present as a shoulder of the imine band. Furthermore, the absence of the vibration band characteristic of the aldehyde group (around 1661 cm^−1^) in the FTIR of xerogels supported the total conversion of the aldehyde into imine units. The changes that occurred between 3700 and 3000 cm^−1^ spectral region in the spectra of the xerogels in comparison with chitosan indicate modifications in terms of hydrogen bond environment due to the imination with 5-methoxysalicylaldehyde.

All these data supported the successful reaction of the amine units of chitosan with the **A** aldehyde.

**Figure 2:**
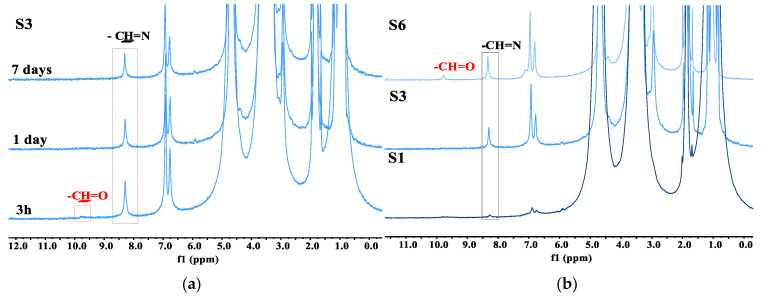
(**a**) ^1^H-NMR spectra of the hydrogel (**S3**) over time; (**b**) ^1^H-NMR spectra of the studied hydrogels after 7 days.

Given the well-established reversibility of imine formation influenced by factors such as water content, dilution, pH, aldehyde reactivity, and imine stability, the process was monitored using ^1^H-NMR spectroscopy. As shown in Figure 2a and Figure S1a, the characteristic imine proton signal appeared as a singlet at around 8.3 ppm in the ^1^H-NMR spectra of **S1**, **S3**, and **S6** hydrogels [30,31,32,33,34,35,36,37,38,39]. The persistent aldehyde proton signal at around 9.8 ppm during the initial 3 h post-gelation indicated incomplete aldehyde conversion to imine units, consistent with the reversible nature of imine formation in aqueous environments (Figure 2b) [40,41]. Time-dependent NMR analysis of the CH=N/CHO integral ratio (Figure S1b) revealed a gradual shift in equilibrium toward imine formation, ending in a stable maximum conversion of 81% for **S6** after seven days. Notably, **S1** and **S3** underwent almost complete conversion within one day, suggesting that higher aldehyde concentrations promote increased imine formation.

**Scheme 3:**
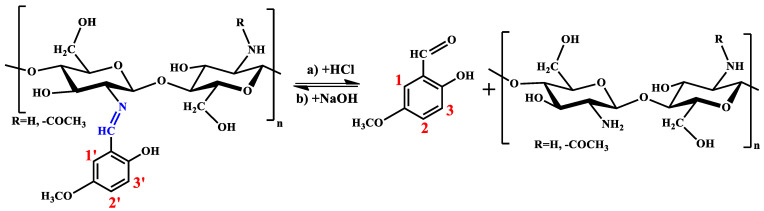
Schematic representation of pH–responsiveness of the hydrogels under different pH conditions.

**Figure 3:**
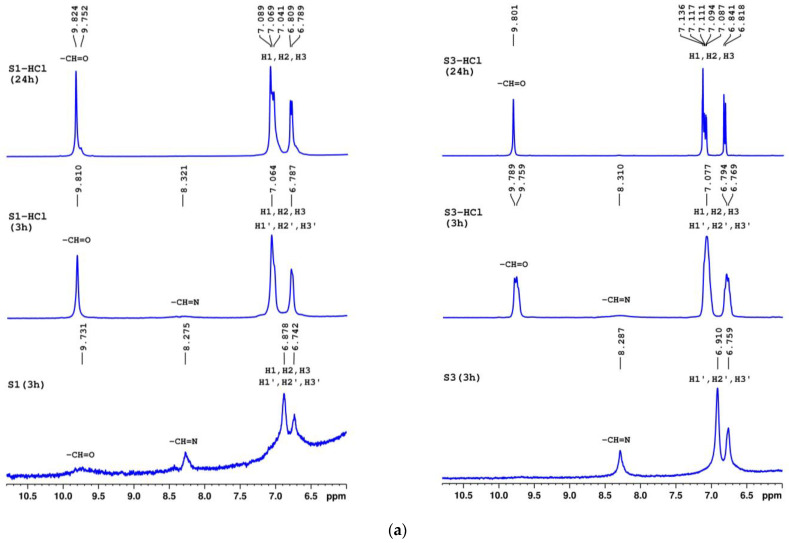

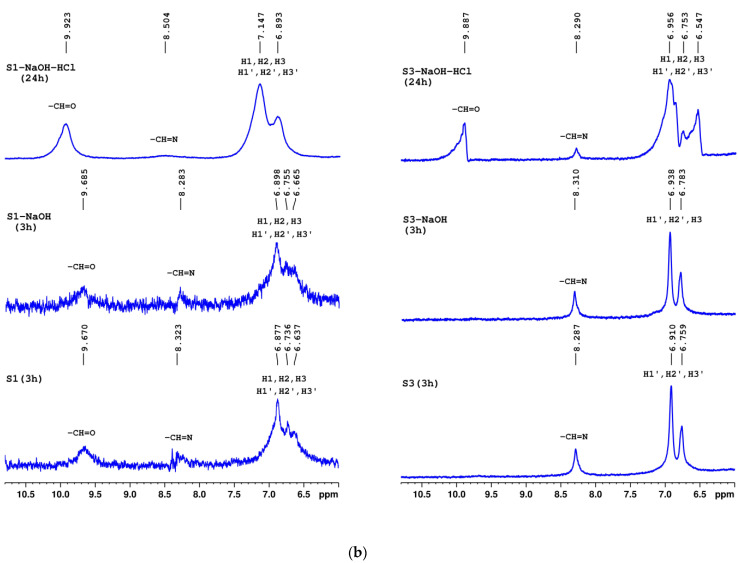
^1^H NMR spectra of representative hydrogels (**S1** and **S3**) obtained under different pH conditions over time: (**a**) acidic pH and (**b**) basic pH.

Further, to evaluate the *pH*–*responsiveness and stability of imine linkages* within hydrogels, samples **S1** and **S3** were subjected to two distinct pH conditions (Scheme 3). In the *first experiment*, hydrogels were exposed to HCl to induce acidic conditions. In the second one, the hydrogels were initially treated with NaOH, followed by neutralization with HCl. NMR analysis revealed a dynamic behavior of the imine bonds (Figure 3). The appearance of a proton signal attributed to aldehyde groups upon HCl treatment indicated the shifting of imination equilibrium to the reagents according to the reversibility of imine formation (Figure 3a). Conversely, the addition of NaOH impacted a slight increase of the imine protons signal, suggesting the shifting of imination equilibrium to the products, i.e., stabilization under alkaline conditions (Figure 3b). Subsequent acidification reversed this trend, confirming the pH–dependent equilibrium of the imine reaction [42]. These observations confirmed the pH–sensitivity of the developed hydrogels.

### 2.2. Morphology of the Hydrogels

**Figure 4:**
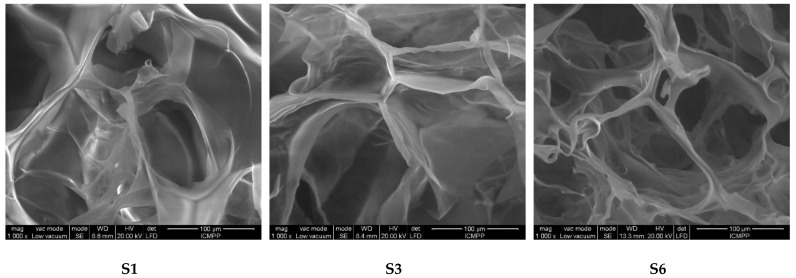
SEM micrographs of **S1**, **S3**, and **S6** hydrogels.

The morphology of the xerogels was investigated by performing scanning electron microscopy. As it could be observed, all samples exhibited a highly porous structure with approximately 100 µm diameter pores. While sample **S6** showed a fibrous appearance, samples **S1** and **S3** displayed interconnected pore networks (Figure 4). The observed morphological differences between the samples can be attributed to variations in terms of crosslinking density. A lower crosslinking density results in longer segments of ‘free’ chitosan chains between two crosslinking points, leading to a looser network characterized by thin pore walls.

Under polarized light (Figure S2), pronounced birefringence and a striated texture, particularly evident in **S1**, suggested a smectic architecture [43,44], attributable to the supramolecular ordering of the imine units into layers, supporting the formation of imine clusters as crosslinking nodes.

### 2.3. Swelling Studies of 5-Methoxysalicyl-Imine-Chitosan Hydrogels (**Sx**)

**Figure 5:**
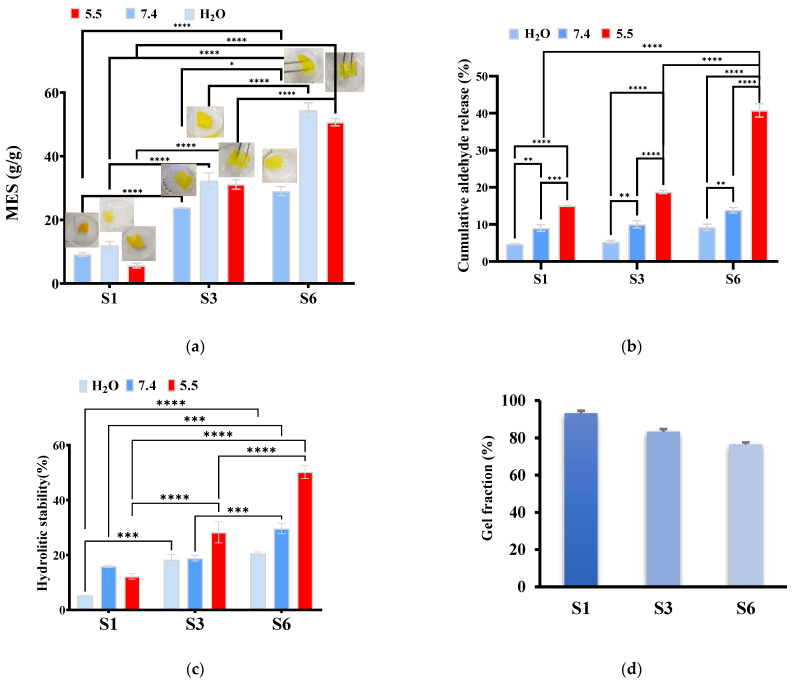
(**a**) Mass equilibrium swelling (**MES**) of hydrogels as a function of pH and representative images of hydrogels after swelling in different media; (**b**) cumulative aldehyde release from xerogels in different media (*: *p* < 0.05; **: *p* < 0.01; ***: *p* < 0.03; ****: *p* < 0.0001); (**c**) hydrolytic stability of xerogels in different media (***: *p* < 0.03; ****: *p* < 0.0001); (**d**) gel fraction of the understudy xerogels (**Sx**).

With the aim to assess the influence of the reversibility of imine linkages on the swelling properties of hydrogels, the swelling behavior was investigated in media with different pHs (pH 7.4, 5.5, and neutral pH). It is known that the stability of the imine linkages is influenced by the pH of the medium, an increase in acidity favoring the shifting of the imination equilibrium to the reagents [45,46,47]. Moreover, with the pH decrease, there is competition in the dissolution and swelling process, which can lead to an increase in the swelling rate but also a decrease in their stability. In our case, all samples swelled in all pH media, reaching equilibrium within 3 h or seven days (Figure S3). The mass equilibrium swelling (**MES**) of the hydrogels increased from 9 to 29 (g/g) in physiological pH media (pH = 7.4), from 6 to 51 (g/g) in acidic media (pH = 5.5), and from 12 to 55 (g/g) in water, respectively (Table S1). From Figure 5a, it can be observed that the swelling ratio increased with a decrease in the crosslinking density, which correlates well with the SEM data. Samples with lower or medium crosslinking (**S3** and **S6**) had a loose network of pores and fibers, leading to higher swelling compared to those with higher crosslinking density (**S1**), which had interconnected pores.

Also, hydrogels exhibited significantly higher swelling ratios in water (neutral pH) compared to acidic buffer solutions (Figure 5a), particularly for the lower crosslinked sample **S6**. This enhanced swelling in water is attributed to the presence of free amino groups (NH_2_) in chitosan. At neutral pH, these groups become ionized, leading to strong electrostatic repulsion between the positively charged groups. This repulsion disrupts hydrogen bonds within the hydrogel network, creating a more open and expanded structure that allows for greater water absorption [48]. Conversely, in a more acidic environment (lower pH), protonation of the amino groups happens, and while this also creates charged groups, the overall electrostatic repulsion might be weaker compared to the neutral case. Additionally, acidic environments can promote stronger hydrogen bonding between other components of the hydrogel network. These combined effects (potentially weaker repulsion and stronger hydrogen bonding) can lead to a denser hydrogel structure and reduced swelling, as evidenced by the lower **MES** values observed in Figure 5a for acidic media compared to water [49].

**Figure 6:**
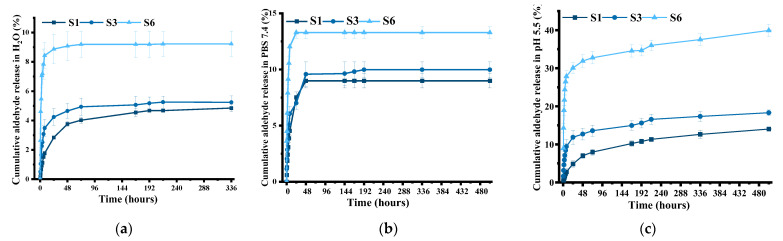
Cumulative aldehyde release from xerogels in different media over time; (**a**) H_2_O; (**b**) PBS (pH = 7.4); (**c**) acetate buffer (pH = 5.5).

Apart from the media nature (buffer vs. water), this behavior can be attributed to a competition between swelling and dissolution. In acidic media, the imination equilibrium shifts toward the reagents, leading to partial hydrolysis of the imine linkages within the hydrogel. As the imine linkages partially hydrolyze, the crosslinking density decreases. Chitosan, being more prone to dissolution, experiences weakening of its network structure. This weakening contributes to the observed erosion of the sample after 21 days. For samples **S1** and **S3** (with higher crosslinking), the swelling and dissolution effects are less pronounced. Statistically significant lower **MES** values for these samples support this observation (Figure 5a). Furthermore, UV analysis revealed that the rate of aldehyde release was significantly higher in acidic swelling media compared to neutral conditions (Figure 5b and Figure 6, Table S2).

In media of physiological pH, the swelling degree was the lowest, likely due to a balance of electrostatic interactions and hydrogen bonding within the hydrogel network, as other authors reported [50,51]. No discolorations or erosions were observed in the hydrogels, and UV analysis indicated lower aldehyde release, suggesting excellent hydrogel stability in physiological media (Figure 5b and Figure 6).

Xerogel stability is affected by both the crosslinking degree and pH (Figure 5c). As anticipated, sample **S1**, which had the highest crosslinking degree, exhibited the greatest hydrolytic stability. Conversely, sample **S6** displayed the lowest stability, especially under acidic conditions. These observations align with the hydrogel’s swelling capacity and aldehyde release in various media [52].

Furthermore, the crosslinking degree of the obtained hydrogels was estimated by correlating this parameter with the gel fraction of the understudied hydrogels. It was observed that the gel fractions of the **Sx** increased with the increase of the amount of aldehyde used in hydrogels’ obtaining (Figure 5d), in agreement with the swelling data according to which, the xerogels obtained using lower amounts of aldehyde were able to absorb higher amounts of water due to their looser network.

The dynamic character of the xerogels, attributed to the reversibility of imine linkages, was further assessed by investigating the release of 5-methoxysalicylaldehyde (**A**) in the same media in which they were swelled (Table S2). The rate of aldehyde release exhibited a strong correlation with both pH and the degree of crosslinking within the xerogels (Figure 5b and Figure 6). The aldehyde release proceeds in two steps: (i) *a burst release* within 8 h, followed by (ii) a progressive release over the next 14 days (Figure 6).

This observation reinforces the hypothesized influence of imine reversibility on the release process. The burst release was mainly attributed to the free, non-covalently bonded aldehyde, which easily diffused through the hydrogel. Further, the slower release was attributed to the progressive shifting of the imination equilibrium to the reagents to counteract the aldehyde removal by dissolution in the aqueous media [53,54,55]. Additionally, a decrease in the crosslinking degree resulted in a higher percentage of released aldehyde, especially in the first stage (**S6**) (Figure 6). This correlates well with ^1^H-NMR studies, which showed higher amounts of free aldehyde for a lower amine/aldehyde ratio. Moreover, a lower amount of aldehyde in hydrogel will induce lower concentrations of aldehyde in the release medium, which is favorable for the shifting of the imination equilibrium towards starting materials. For a more exhaustive interpretation, a mathematical analysis of the in vitro release profiles was conducted, consisting of fitting the data obtained for the two release stages on five mathematical models: zero order, first order, Higuchi, Korsmeyer–Peppas, and Hixson–Crowell. The accuracy of fitting was assessed by calculation of the squared correlation coefficient (R^2^) (Table 1a–c) [56]. The data comparison brought to light interesting conclusions, as follows:

From the fitting of the release data in the first stage (0.5–8 h) (Table 1a–c and Figure S4), it can be seen that almost all the samples gave a high correlation coefficient in all media (R^2^ = 0.92–0.99), indicating a complex release mechanism involving diffusion and swelling processes. The Korsmeyer–Peppas model provided the best fit, allowing the determination of kinetic constant (K) and release exponent (n) values through linear regression. For samples **S1** and **S3**, n values ranged from 0.68 to 0.82, suggesting anomalous (non-Fickian) diffusion during the *burst stage*. In contrast, sample **S6** exhibited n values between 0.39 and 0.44, indicative of Fickian diffusion [57]. Swelling experiments corroborate the observed release profiles: sample **S6** exhibited rapid swelling to a high maximum equilibrium swelling (9–41 g/g), while samples **S1** and **S3** displayed slower swelling with lower **MES** values (from 4 to 19 g/g) (Table S1). These findings also align with the established understanding that swelling can significantly influence release kinetics by increasing matrix porosity, expanding the surface area available for molecular interactions, and facilitating diffusion through enlarged pathways within the hydrogel network [52].

The excellent fitting of the Higuchi model (R^2^ = 0.96, 0.99), confirmed that diffusion plays the principal role in the aldehyde release. Furthermore, considering that in the first stage, the aldehyde’s concentration in the release medium was much lower than the saturation concentration, it can be assumed that the reversible covalent bonding of **A** to chitosan prohibited its fast dissolution and promoted its slow diffusion through the hydrogel along the imination equilibrium shifting. Furthermore, the diffusion process should be retarded by the possibility to form physical forces with the chitosan [58,59].

In the case of zero order, first order, and Hixson–Crowell models, the fitting was good, confirming that swelling, content of **A,** and dissolution velocity played a secondary role in its release in the first four hours.

The correlation coefficients for the second release stage were low for all samples (Table S3 and Figure S4). This observation aligns with the potential dominance of matrix erosion on aldehyde release during this extended timeframe. Additionally, sample **S6** in PBS exhibited near-complete release of the aldehyde after 24 h. As previously mentioned, the presence of more amine groups in **S6** could introduce electrostatic repulsion between the xerogel chains. This repulsion may increase swelling, leading to increased matrix porosity and facilitating faster release of the entrapped aldehyde. Additionally, significant swelling in **S6** could have contributed to matrix erosion. The formation of additional release channels due to erosion might further accelerate aldehyde release in the second stage [48].

### 2.4. The Antioxidant Activity of the Hydrogels 

Literature data show that chitosan has antioxidant activity due to the ability of hydroxyl and amine units to bind free radicals. This is why the antioxidant activity will depend on the active surface of the chitosan materials and mobility of the chitosan chains; chitosan in its solid state displays less antioxidant activity compared with chitosan [60]. Furthermore, the aldehyde crosslinker possesses a hydroxyl group with the potential to inhibit radicals too. We hypothesized that the reversible nature of the imine bonds will promote a continuous aldehyde release under the pressure of external stimuli, leading to a dynamic long-term antioxidant hydrogel. To this aim, a three-stage experiment was designed.

**Figure 7:**
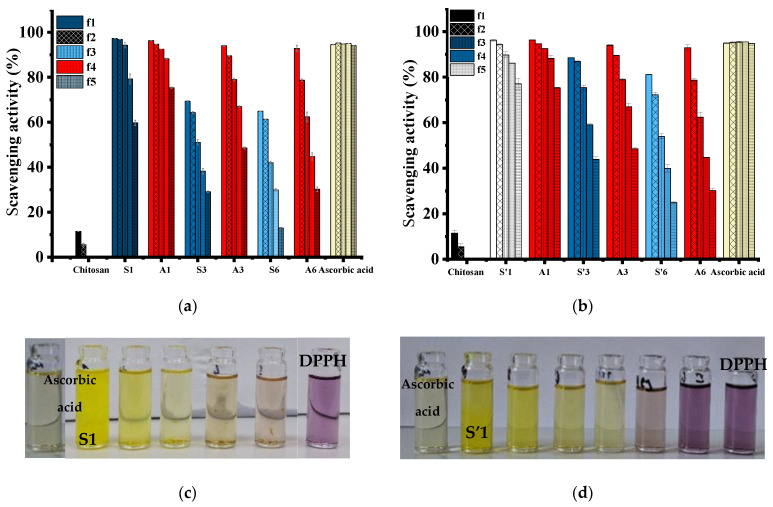
Antioxidant activity of hydrogels: (**a**) **Sx** and (**b**) **S’x** and their references; visual representation of the colorimetric changes in hydrogel solutions at different concentrations following exposure to DPPH: (**c**) **S1** and (**d**) **S’1**.

In the first stage, the antioxidant activity of the hydrogels was determined compared to chitosan and aldehyde references at similar concentrations, compared to ascorbic acid (Figure 7a and Figure S5). The measurements were conducted on solutions prepared by serial dilution in water (f1–f5 dilutions). While chitosan showed very low antioxidant capacity even for higher concentrations, the aldehyde displayed activity almost similar to ascorbic acid, which is considered a standard. The hydrogel activity was slightly diminished compared to aldehyde precursor, yet the difference was statistically significant, a fact attributed to the loss of mobility by partial binding of aldehyde to chitosan chains via imine bonds within the hydrogel network (Figure 7).

**Figure 8:**
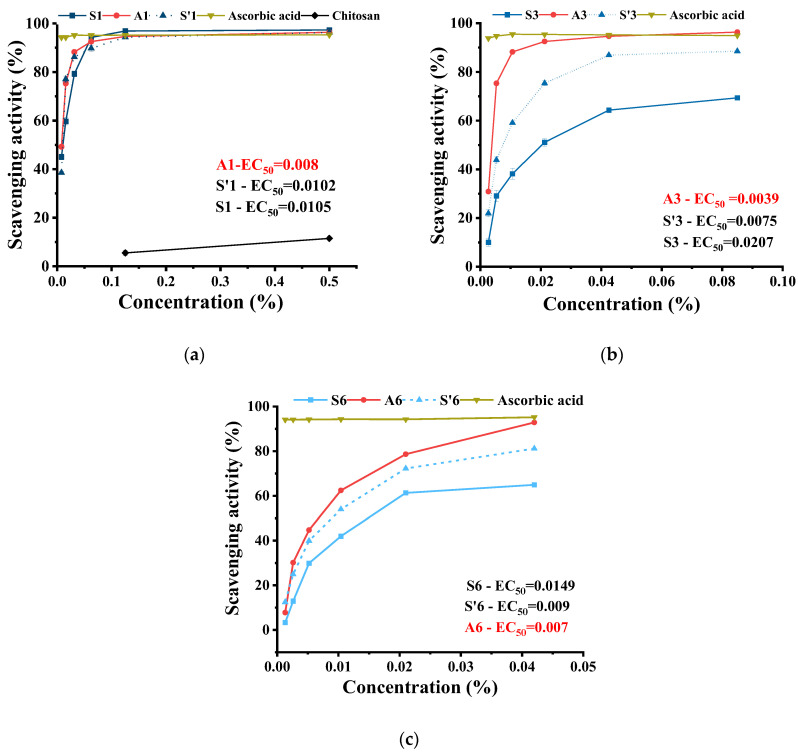
The scavenging activity of the hydrogels: (**a**) **S_1_**, **S’_1,_** aldehyde (A_1_), chitosan, and ascorbic acid; (**b**) **S_3_**, **S’_3_**, aldehyde (A_3_), chitosan, and ascorbic acid; (**c**) **S_6_**, **S’_6_**, aldehyde (A_6_), chitosan, and ascorbic acid.

This reversible character of the imine linkage likely causes a gradual release of the active component, resulting in a slower scavenging effect on free radicals. As expected, hydrogel **S1**, containing the largest amount of aldehyde crosslinker, displayed the highest antioxidant activity even at low concentrations (Figure 8a). Similarly, hydrogels **S3** and **S6** showed the highest activity up to a dilution factor of c4 (Figure 8b,c). However, a decrease in activity was observed at higher dilutions, leading to EC_50_ values from 10% for **S1** to almost 15% for **S6**.

In the second stage of the experiment, for a more accurate attribution of the antioxidant activity, the hydrogels (**S’x**) were prepared in more diluted chitosan (0.66% compared to 2%), whose NMR indicated a lower conversion of aldehyde to imine bonds (Figure 8 and Figure S6), and the scavenging activity was measured for similar concentrations as those used in the first stage. The antioxidant activity showed statistically significant higher values compared with the first set of measurements, with a decrease of EC_50_ up to 7.5% (Figure 8 and Figure S5). Considering the differences in imination degree between the two sets of samples, it can be assumed that the free aldehyde is the main promoter of antioxidant activity. It can be envisaged that there is a relationship between the antioxidant activity of the hydrogels and the imination degree.

**Figure 9:**
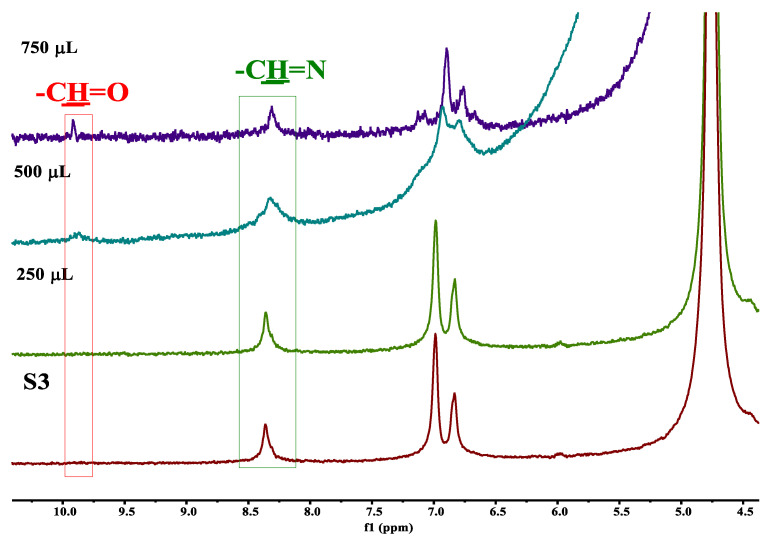
^1^H-NMR spectra of hydrogel **S3** after exposure to different amounts of DPPH.

In the third stage, we investigated the influence of DPPH on the reversibility of imine linkages. Our hypothesis was that the gradual release of aldehyde groups from imine units, facilitated by the DPPH–mediated cleavage, would enhance the antioxidant capacity of the hydrogels. To test this, ^1^H-NMR spectra of hydrogel **S3** were acquired after incubating the samples with various amounts of DPPH concentrations (0.188 mM) (Figure 9). As anticipated, a clear correlation was observed between increasing DPPH concentration and a decrease in signal intensity of the imine proton, accompanied by the appearance of aldehyde proton signals. These findings support our hypothesis, indicating that the dynamic nature of imine linkages, coupled with aldehyde-mediated antioxidant activity, contributes significantly to the hydrogel’s overall antioxidant properties.

## 3. Conclusions

This study investigated the influence of reversible imine linkages on the properties of three hydrogels prepared by the acid condensation reaction of chitosan with 5-methoxysalicylaldehyde. FTIR and NMR analyses confirmed that hydrogelation occurs through the formation of imine units on chitosan, which self-assemble into crosslinking clusters. These hydrogels exhibited porous morphology, pH–responsive swelling behavior, and enhanced antioxidant activity compared to unmodified chitosan. The reversible nature of the imine linkages facilitated the gradual release of 5-methoxysalicylaldehyde, contributing to their antioxidant properties. The rate of aldehyde release exhibited a strong correlation with both pH and the degree of crosslinking within the xerogels. As expected, the aldehyde release proceeds in two steps: (i) a burst release within 8 h, followed by (ii) a progressive release over the next 14 days. This suggests a combination of diffusion and matrix erosion mechanisms. The progressive release of aldehyde induced long-term antioxidant activity. These findings emphasize the potential of these dynamic hydrogels for various biomedical applications, particularly those requiring pH–responsive behavior and controlled release of bioactive molecules.

## 4. Materials and Methods

### 4.1. Materials

The materials employed in this study were procured from Sigma Aldrich (Darmstadt, Germany) and utilized without additional purification: low molecular weight chitosan (M_w_ = 198 kDa), 5-methoxysalicylaldehyde (**A**) (98% purity), ethanol (99.8%), and glacial acetic acid (99%). The chitosan’s average viscometric molecular weight (Mw) was determined through viscosimetry (Mw = 198 kDa), while its deacetylation degree (DA = 82%) was calculated using ^1^H-NMR spectroscopy (Figures S7 and S8). Acetate buffer solution (pH = 5.5) and phosphate buffer (PBS) solutions at pH 7.4 were prepared in our lab, following previously outlined methods [28]. Bidistilled water was also obtained in our laboratory.

### 4.2. Synthesis of 5-Methoxysalicyl-Imine-Chitosan Hydrogels (Sx)

The synthesis was carried out through an acid condensation reaction between chitosan and 5-methoxysalicylaldehyde (**A**), adapting a previously reported method for other imino-chitosan derivatives (Scheme 1a) [30]. Briefly, 0.120 g of chitosan (corresponding to 5.8 × 10^−4^ mmol glucosamine repeating units) was dissolved in 6 mL of acidic water (0.7% acetic acid solution: 42 μL acetic acid in 6 mL ultrapure water) under vigorous magnetic stirring (750 rpm). Subsequently, the chitosan solution (2%) was heated to 55 °C, and a 2% solution of aldehyde (**A**) in ethanol was gradually added. The volume of the aldehyde (**A**) solution was adjusted to achieve three different molar ratios of NH_2_ and CHO functional groups: 1/1, 3/1, and 6/1 (Scheme 1b). The resulting reaction mixture was maintained at 55 °C under vigorous magnetic stirring until the viscous solution transformed into a soft material that passed the inverted tube test, indicating hydrogel formation. A clear correlation between the NH_2_/CHO molar ratio and gelation time was observed, with gelation occurring immediately (1 min) for the 1/1 ratio, more slowly (30 min) for the 3/1 ratio, and very slowly (7 days) for the 6/1 ratio (Table 1). The ethanol was subsequently removed by leaving the vials uncovered until the initial volume of the chitosan solution was reached. The obtained hydrogels (noted **Sx**, where x represents the NH_2_/CHO molar ratio) appeared as transparent, deep yellow, semi-solid materials with a smooth texture (Scheme 1b). Their corresponding solid materials (xerogels) were prepared by lyophilization of the hydrogels. Additionally, the possibility that the aldehyde would react with ethanol was investigated by NMR (Figure S9). The mixture was kept at 55 °C for 3 h, following the same experimental protocol as in the case of the hydrogels. The NMR spectrum did not present any chemical shifts besides the ones corresponding to the aldehydes and ethanol.

### 4.3. Characterization

#### 4.3.1. Xerogels

Xerogels were prepared by immersing the sample in liquid nitrogen, followed by lyophilization using a Labconco Free Zone Freeze Dry System (Québec City, QC, Canada) at −52 °C and 1.510 mbar for 48 h.

#### 4.3.2. NMR Spectroscopy

H-NMR spectra were acquired using a Bruker Avance NEO 400 MHz Spectrometer (Bruker BioSpin, Ettlingen, Germany), equipped with a 5 mm QNP direct detection probe and z-gradients. For NMR analysis, hydrogels were prepared in NMR tubes using deuterated water, ethanol, and glacial acetic acid. Chemical shifts are reported as δ values (ppm) relative to the residual peak of deuterated water.

#### 4.3.3. Fourier–Transform Infrared (FTIR) Spectroscopy

Fourier–Transform Infrared (FTIR) spectroscopy was employed for analysis. ATR–FTIR spectra were collected using a Bruker Vertex 70 Ettlingen FTIR spectrometer (Bruker Optik GmbH, Ettlingen, Germany). Spectra were recorded within the 600–4000 cm^−1^ range, accumulating 32 scans at a resolution of 4 cm^−1^.

#### 4.3.4. The Scanning Electron Microscopy (SEM)

To characterize hydrogel morphology, thin sections of the corresponding xerogels were examined with Verios G4 UC Scanning Electron Microscope (SEM) (FEI Company, Hillsboro, OR, USA) type Quanta 200, coupled with an energy dispersive spectrometer (EDS, EDAX Octane Elite) (FEI Company, Hillsboro, OR, USA) for determination of elemental composition.

#### 4.3.5. The Polarized Light Microscopy (PLM)

To investigate the supramolecular structure of the hydrogels, thin sections of xerogels were examined under Polarized Light Microscopy using a Zeiss (Carl Zeiss Microscopy GmbH, Oberkochen, Germany). Axio Imager.A2m microscope equipped with an Axiocam 208cc camera (Carl Zeiss Microscopy GmbH, Oberkochen, Germany).

#### 4.3.6. The Cumulative Aldehyde Releases

5-methoxysalicylaldehyde (**A**) release from hydrogels was monitored using UV–Vis spectroscopy on an Agilent Cary 60 spectrophotometer (Agilent Technologies, Inc. Headquarters, Santa Clara, CA, USA). Xerogels (20 mg) were incubated in 10 mL of release medium (ultrapure water, PBS). At predetermined intervals, 2 mL of supernatant was replaced with fresh medium. The absorbance of the collected supernatants was measured at 258.87 nm, corresponding to the aldehyde’s absorption band. The concentrations of the aldehyde were calculated using a calibration curve previously drawn using solutions of 5-methoxysalicylaldehyde of known concentrations. Cumulative aldehyde release (*A*) was calculated using the following Equation (1):

(1) $$A(\%)=[\frac{\left(10Cn+2\Sigma Cn-1\right)}{mo}]\times 100$$

where C_n_ and C_n−1_ are aldehyde concentrations after n and n − 1 sampling, respectively, and m_o_ is the initial aldehyde content in the xerogels. The release kinetics were plotted as cumulative aldehyde release as a function of time. To understand the mechanism of aldehyde release from the hydrogels, five mathematical models were employed: zero order, first order, Korsmeyer–Peppas, Higuchi, and Hixson–Crowell models [56] (Table 2).

#### 4.3.7. Swelling Behavior and Stability of the Xerogels in Media with Different pH

To assess swelling behavior, approximately 20 mg of xerogel samples (dimensions: 1 cm × 1 cm × 0.3 cm) (wd) were immersed in 10 mL of distilled water or buffer solutions (pH 5.5 and 7.4). Samples were periodically weighed until a constant mass (ws) was reached. The mass equilibrium swelling (**MES**) was calculated using the following Equation (2):

(2) $$MES=\frac{ws-wd}{wd}$$

Furthermore, xerogel stability was evaluated by determining mass loss in the corresponding swelling media after 21 days. The supernatant was removed after this period, and the swollen samples were washed three times with ultrapure water, lyophilized, and weighed (wf). Mass loss (ML) was calculated using the following Equation (3):

(3) $$ML(\%)=\frac{wf-wd}{wd}\times 100$$

#### 4.3.8. The Antioxidant Activity

Antioxidant activity of the hydrogels was performed by DPPH–Radical Scavenging Assay method, on six serial diluted samples: 3×, 6×, 12×, 24×, 48×, and 96× in 0.7% acetic acid solution. For example, for the 3× dilution, 1 mL hydrogel **S1**, **S3**, and **S6,** respectively, was dispersed in 2 mL acetic acid aqueous solution. From the resulting dispersion, 1.5 mL was mixed with 1.5 mL DPPH solution in ethanol (0.025 mg/mL), vortexed, incubated in dark at room temperature for 1 h, and then subjected to UV-Vis (Agilent Cary 60 UV–Vis spectrophotometer, Agilent Technologies, Inc. Headquarters, Santa Clara, CA, USA) measurements by reading the DPPH absorbance at 517 nm. The radical scavenging ability (RSA) was determined with the following Equation (4):

(4) $$RSA=\frac{A_{r}-A_{s}}{A_{r}}\times 100$$

where A_r_—the absorbance of the DPPH solution used as reference; A_s_—the absorbance of the DPPH solution incubated with the hydrogel samples. Then, the obtained RSA values were plotted vs. concentration, and concentration showing 50% scavenging activity (EC_50_) was determined.

#### 4.3.9. Gel Fraction

The gel fraction was determined according to a similar method described in the literature with some modifications. Pre-weight xerogels pieces (mi) (30 mg) were immersed in sufficient amounts of ultrapure water for 4 h. After that, the water was removed, and the samples were dried at 60 °C under vacuum for 48 h before being weighted (mf). The gel fractions were calculated using the following Equation (5):

(5) $$Gel\;fraction\;(\%)=\frac{mf}{mi}\times 100$$

#### 4.3.10. Statistics

All experiments were conducted in triplicate. Data are presented as mean ± standard deviation (SD). Statistical differences between groups were assessed using one-way ANOVA with GraphPad Prism or SPSS version 23.0. Differences were considered statistically significant at *p* < 0.05.

## Figures and Tables

**Table 1:** Parameters from fitting mathematical models to the *first stage* of aldehyde release in different pH media: (**a**) H_2_O, (**b**) PBS (pH = 7.4), and (**c**) acetate buffer (pH = 5.5).

	Model	Zero Order		First Order		Higuchi		Korsmeyer–Peppas			Hixson–Crowell	
Code												
First stage		R^2^	K_0_	R^2^	K	R^2^	K_H_	R^2^	K	n	R^2^	K
**(** **a) H_2_O**												
**S1**		0.98	0.21	0.98	−0.002	0.99	0.76	0.99	0.004	0.82	0.98	−0.003
**S3**		0.92	0.38	0.93	−0.004	0.99	1.39	0.98	0.01	0.68	0.93	−0.006
**S6**		0.81	0.69	0.82	−0.007	0.92	2.63	0.96	0.04	0.39	0.82	−0.01
**(b) PBS (pH = 7.4)**												
**S1**		0.96	0.49	0.98	−0.002	0.99	1.79	0.98	0.011	0.69	0.96	−0.008
**S3**		0.95	0.56	0.95	−0.006	0.99	2.07	0.99	0.016	0.61	0.95	−0.009
**S6**		0.96	1.1	0.96	−0.012	0.99	4.03	0.99	0.05	0.45	0.96	−0.02
**(** **c) Acetate buffer (pH = 5.5)**												
**S1**		0.97	0.33	0.98	−0.003	0.99	1.2	0.99	0.006	0.80	0.98	−0.005
**S3**		0.93	0.99	0.94	−0.01	0.99	3.7	0.98	0.026	0.62	0.94	−0.009
**S6**		0.85	2.1	0.87	−0.03	0.96	8.08	0.97	0.11	0.44	0.87	−0.02

**Table 2:** Mathematical models are used for fitting the kinetic experimental data.

Model Name	Equation	Description
**Zero Order**	**Q_t_ = k_o_·t**	**Qt**: amount of aldehyde dissolved, **t**: time, **k_0_**: Zero order release constant
**First Order**	**logQ_t_ = logQ_o_ + k·t/2.303**	**Qo**: initial amount of aldehyde, **Q_t_**: amount of aldehyde released, **t**: time, **k**: first order release constant
**Korsmeyer–** **Peppas**	**M_t_/M_∞_ = K·t^n^**	**M_t_/M_∞_**: fraction of aldehyde released, **t**: time, K: rate constant, n: release exponent
**Higuchi**	**Q_t_ = k_H_·t^1/2^**	**Q_t_**: amount of aldehyde released, **t**: time, **k_H_**: Higuchi dissolution constant
**Hixson–** **Crowell**	**Wo^1/3^ − Wt^1/3^ = k·t**	**Wo**: initial amount of aldehyde, **W_t_**: remaining amount of aldehyde in formulation, **t**: time, **k**: constant

## Data Availability

The data are available on request.

## Supplementary Materials

The following supporting information can be downloaded at: https://www.mdpi.com/article/10.3390/gels10100655/s1, Figure S1: (a) ^1^H-NMR spectra of hydrogels over time; (b) Graphic representation of imine conversion percentage over time; Figure S2: POM micrographs of S1, S3, and S6 hydrogels; Figure S3: Swelling kinetics of hydrogels over time in media with various pH values: (a) H_2_O; (b) PBS (pH = 7.4); (c) Acetate buffer (pH = 5.5) conversion percentage over time; Figure S4: Linear forms of the Korsmeyer-Peppas, Zero Order, Higuchi, Hixson-Crowell, First order models applied for the release of aldehyde from Sx on the first andsecond stage: (a) H_2_O, (b) PBS (pH = 7.4) and (c) Acetate buffer (pH = 5.5); Figure S5: Images of hydrogels solutions (Sx and S’x) and their references obtained after incubation with DPPH solution; Figure S6: (a) ^1^H-NMR spectra of the hydrogels S’x and (b) Graphic representation of imine degree over time; Figure S7: Graph of reduced viscosity vs. chitosan concentration; Figure S8: ^1^H-NMR spectrum of chitosan; Figure S9: ^1^H-NMR spectra of a mixture of aldehyde and ethanol in deuterium oxide, with traces of acetic acid; Table S1: MES values of the xerogels in different media; Table S2: Cumulative aldehyde release (%) in different media; Table S3: Parameters from fitting mathematical models to the second stage of aldehyde release in different pH media: (a) H_2_O, (b) PBS (pH = 7.4) and (c) (pH = 5.5).
