# Supplementary material for: Tuning Antioxidant Function through Dynamic Design of Chitosan-Based Hydrogels
Source: Gels. 2024 Oct 13;10(10):655. doi: 10.3390/gels10100655 (PMC11507920; doi:10.3390/gels10100655)
Supplement: Supplementary file 1 [file gels-10-00655-s001.zip › gels-3231213-supplementary.pdf]

Supporting Information

For

Tuning Antioxidant Function through Dynamic Design of Chitosan-Based Hydrogels

Manuela Maria Iftime [1,*], Gabriela Liliana Ailiesei [1] and Daniela Ailincai [1,2]

[1]"Petru Poni" Institute of Macromolecular Chemistry, Grigore Ghica Voda Alley, 700487 Iasi, Romania
[2] The Research Institute of the University of Bucharest (ICUB), 90 Sos. Panduri, 050663 Bucharest, Romania
[*] Correspondence: ciobanum@icmpp.ro; Tel.: +40-742-008-207

Figure S1. a) ^{1}H-NMR spectra of hydrogels over time; b) Graphic representation of imine conversion percentage over time 2
Figure S2. POM micrographs of S1, S3, and S6 hydrogels 3
Figure S3. Swelling kinetics of hydrogels over time in media with various pH values: a) H_2O; b) PBS (pH=7.4); c) Acetate buffer (pH=5.5) 3
Figure S4. Linear forms of the Korsmeyer-Peppas, Zero Order, Higuchi, Hixson-Crowell, First order models applied for the release of aldehyde from Sx on the first and second stage: a) H_2O, b) PBS (pH=7.4) and c) Acetate buffer (pH=5.5) 4
Figure S5. Images of hydrogels solutions (Sx and S'x) and their references obtained after incubation with DPPH solution 7
Figure S6. a) ^{1}H-NMR spectra of the hydrogels S'x and b) Graphic representation of imine degree over time 8
Figure S7. Graph of reduced viscosity *vs.* chitosan concentration 9
Figure S8. ^{1}H-NMR spectrum of chitosan 10
Figure S9. ^{1}H-NMR spectra of a mixture of aldehyde and ethanol in deuterium oxide, with traces of acetic acid. 11
Table S1. MES values of the xerogels in different media 12
Table S2. Cumulative aldehyde release (%) in different media 12
Table S3. Parameters from fitting mathematical models to the *second stage* of aldehyde release in different pH media: a) H_2O, b) PBS (pH=7.4) and c) (pH=5.5) 12

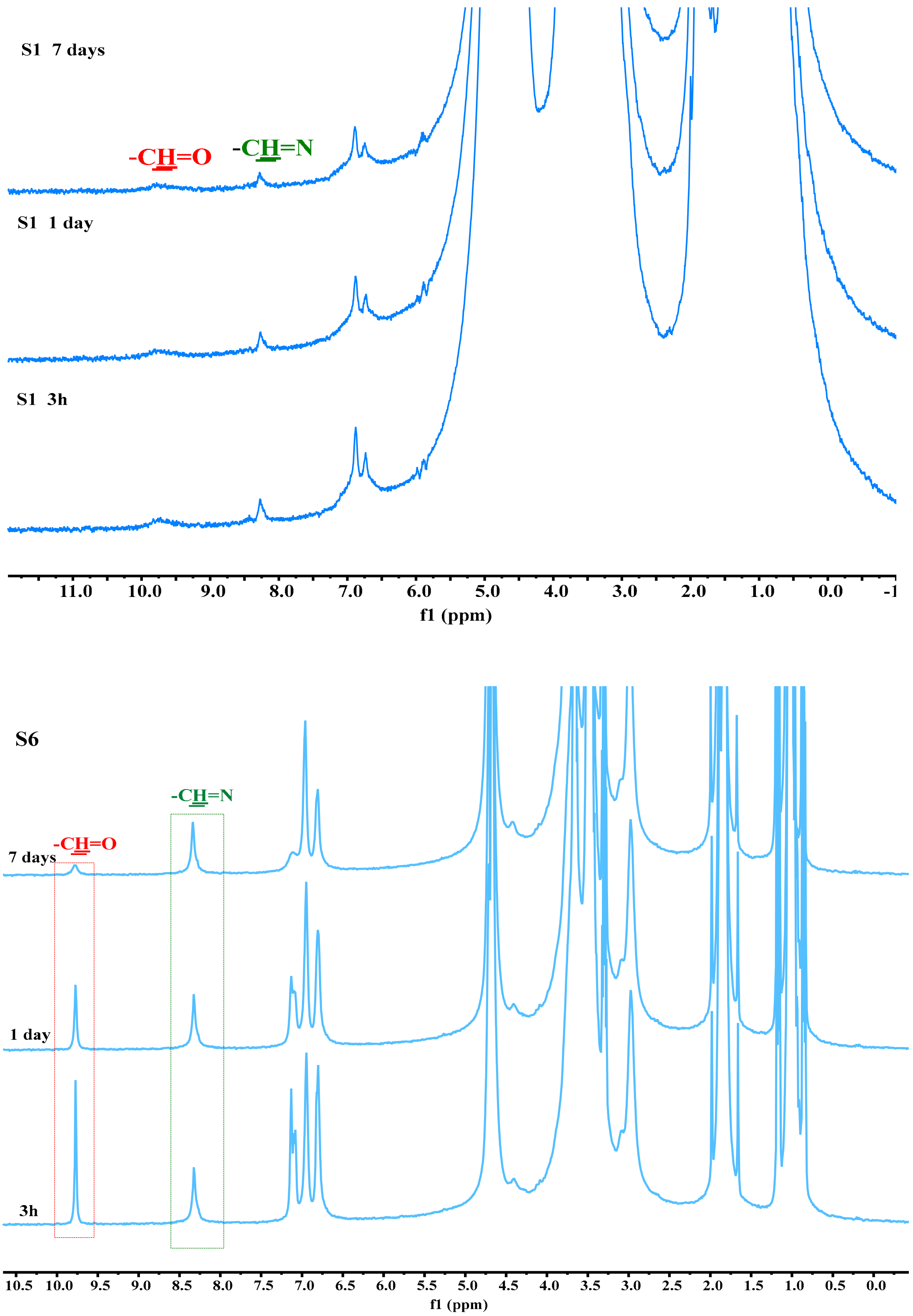
S1 7 days
-CH=O
-CH=N
S1 1 day
S1 3h
11.0 10.0 9.0 8.0 7.0 6.0 5.0 4.0 3.0 2.0 1.0 0.0 -1
f1 (ppm)
S6
-CH=N
-CH=O
7 days
1 day
3h
10.5 10.0 9.5 9.0 8.5 8.0 7.5 7.0 6.5 6.0 5.5 5.0 4.5 4.0 3.5 3.0 2.5 2.0 1.5 1.0 0.5 0.0
f1 (ppm)

a)

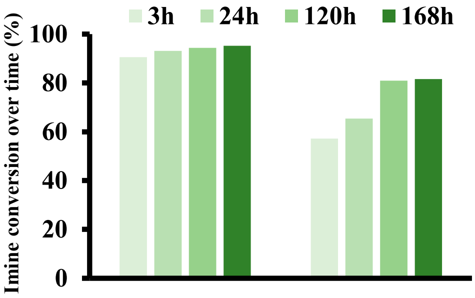

b)

Figure S1. a) ^{1}H-NMR spectra of hydrogels over time; b) Graphic representation of imine conversion percentage over time for S3 and S6 samples

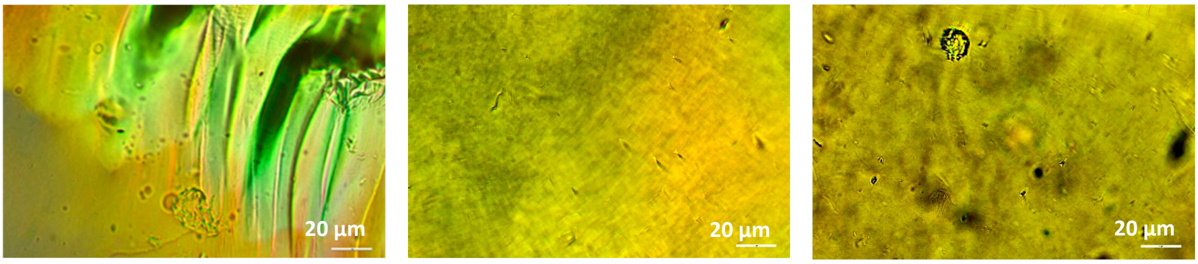

S1 **S3** **S6**

Figure S2. POM micrographs of **S1**, **S3**, and **S6** hydrogels

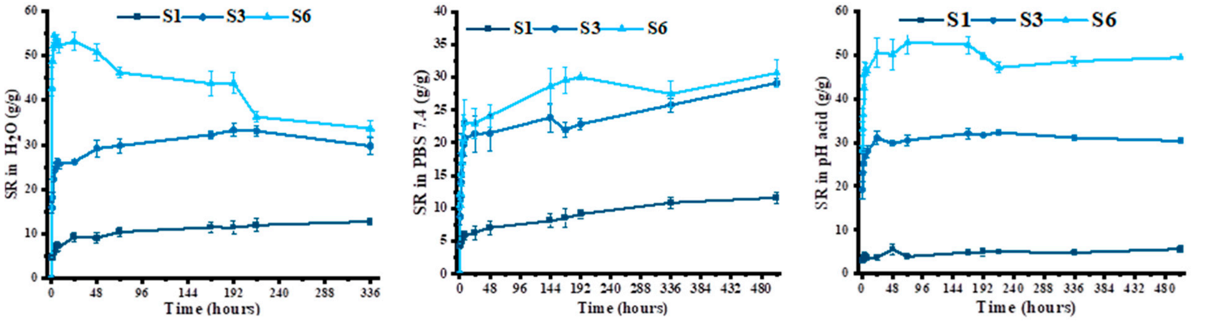

a) **b)** **c)**

Figure S3. Swelling kinetics of hydrogels over time in media with various pH values: a) H_2O; b) PBS (pH=7.4); c) Acetate buffer (pH=5.5).

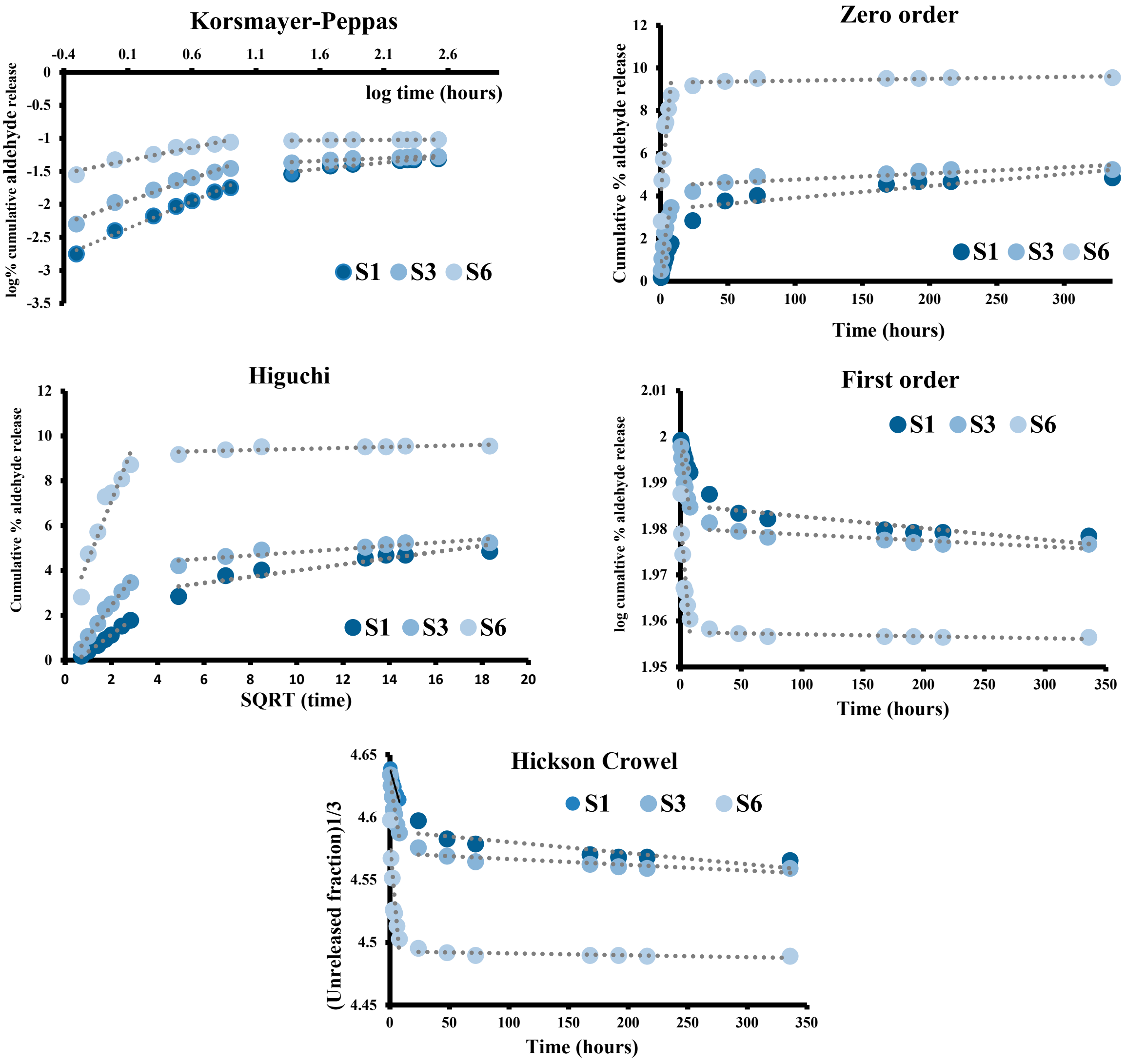

Korsmayer-Peppas
-0.4
0.1
0.6
1.1
1.6
2.1
2.6
log time (hours)
log% cumulative aldehyde release
0
-0.5
-1
-1.5
-2
-2.5
-3
-3.5
S1
S3
S6
Zero order
Cumulative % aldehyde release
12
10
8
6
4
2
0
50
100
150
200
250
300
Time (hours)
S1
S3
S6
Higuchi
Cumulative % aldehyde release
12
10
8
6
4
2
0
2
4
6
8
10
12
14
16
18
20
SQRT (time)
S1
S3
S6
First order
log cumaltive % aldehyde release
2.01
2
1.99
1.98
1.97
1.96
1.95
0
50
100
150
200
250
300
350
Time (hours)
S1
S3
S6
Hickson Crowel
(Unreleased fraction)1/3
4.65
4.6
4.55
4.5
4.45
0
50
100
150
200
250
300
350
Time (hours)
S1
S3
S6

a) H_2O

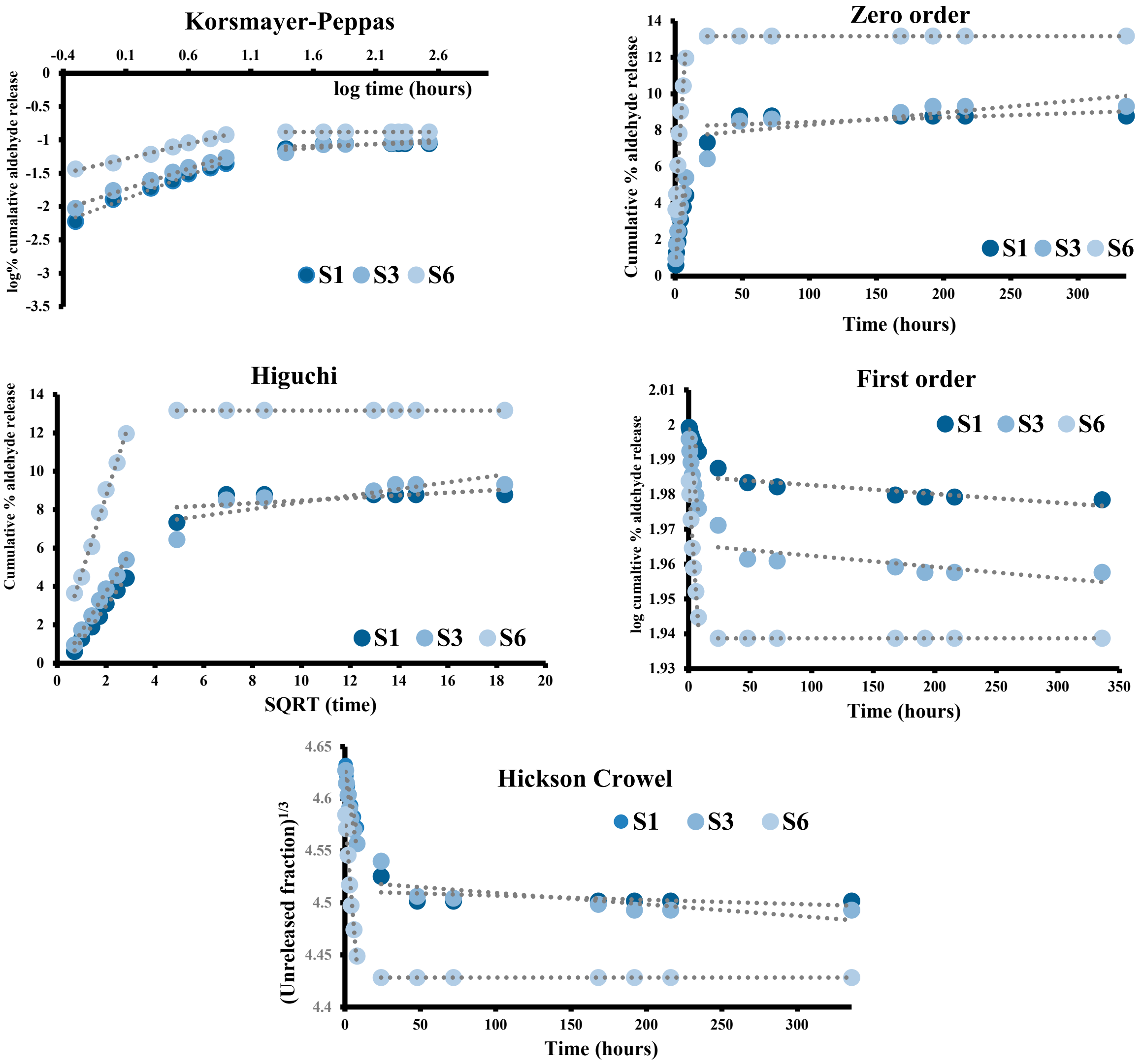
Korsmayer-Peppas
log time (hours)
log% cumalative aldehyde release
S1 S3 S6
Zero order
Cumulative % aldehyde release
Time (hours)
S1 S3 S6
Higuchi
Cumulative % aldehyde release
SQRT (time)
S1 S3 S6
First order
log cumaltive % aldehyde release
Time (hours)
S1 S3 S6
Hickson Crowel
(Unreleased fraction)1/3
Time (hours)
S1 S3 S6

b) PBS (pH=7.4)

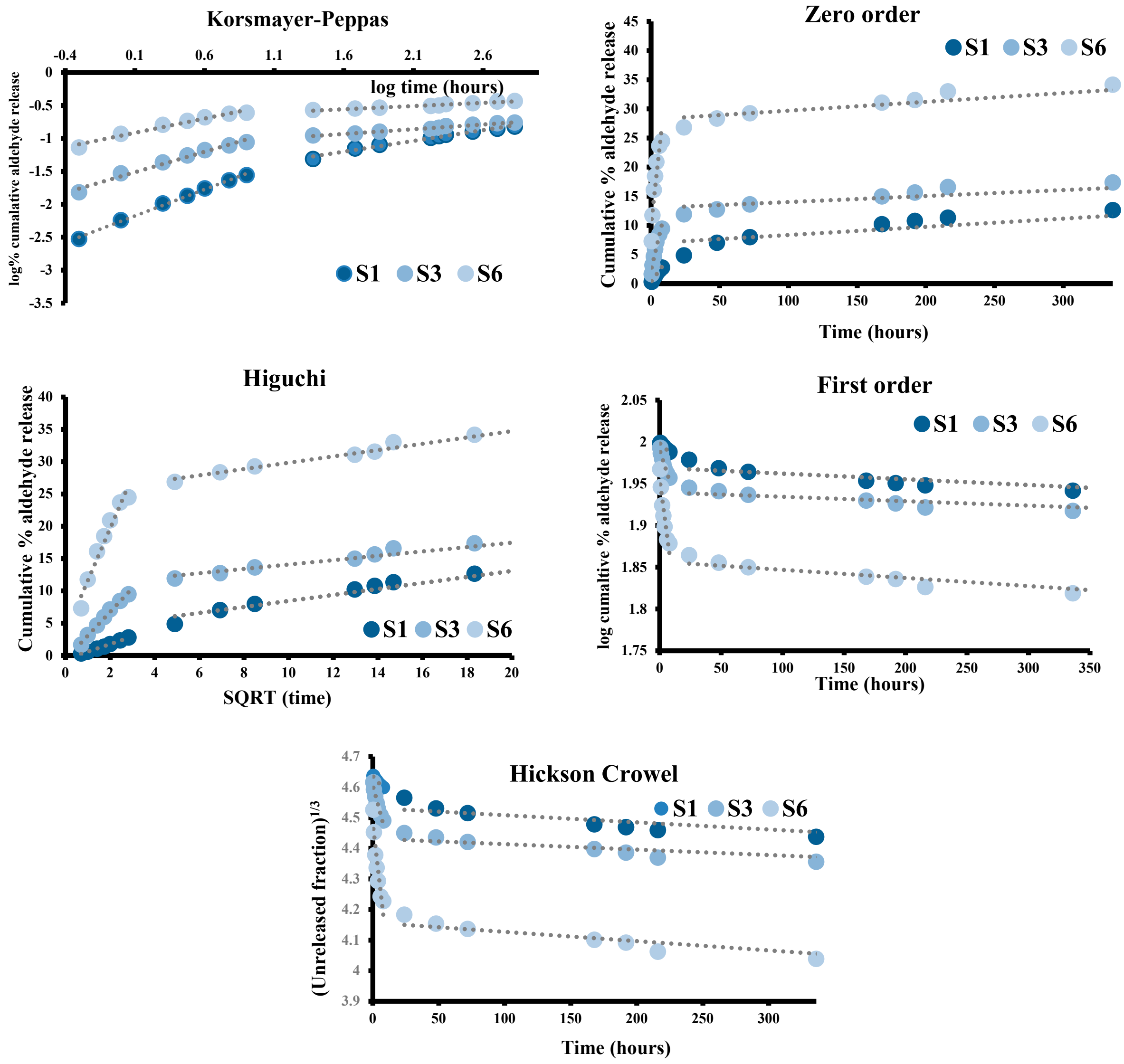

c) Acetate buffer (pH=5.5)

Figure S4. Linear forms of the Korsmeyer-Peppas, Zero Order, Higuchi, Hixson-Crowell, First order models applied for the release of aldehyde from Sx on the *first* and *second* stage: a) H_2O, b) PBS (pH=7.4) and c) acetate buffer (pH=5.5)

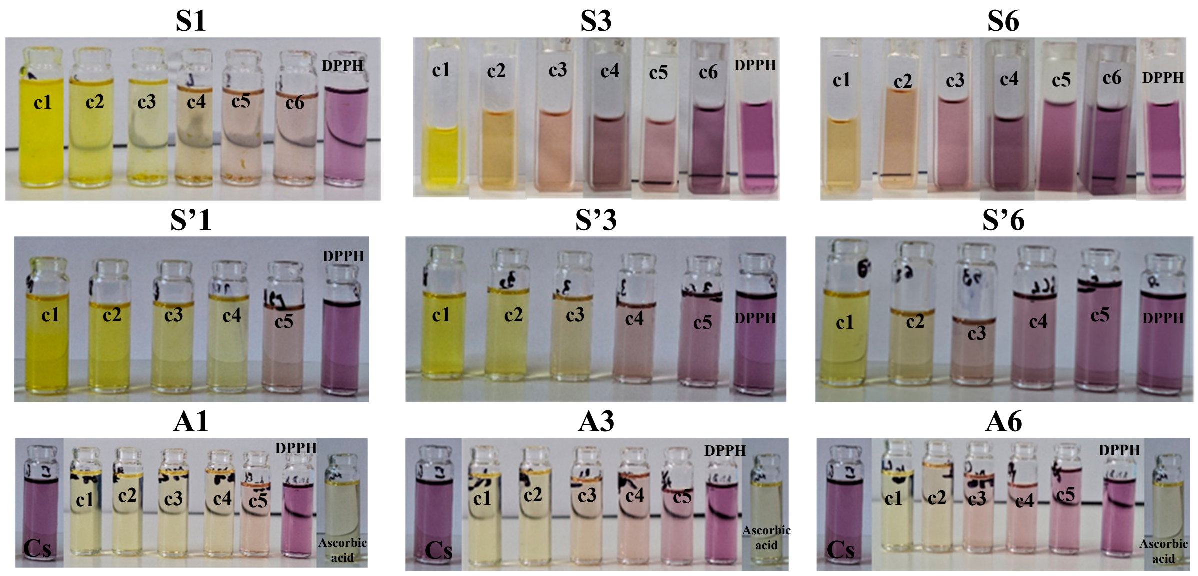

Figure S5. Images of hydrogels solutions (Sx and S’x) and their references obtained after incubation with DPPH solution

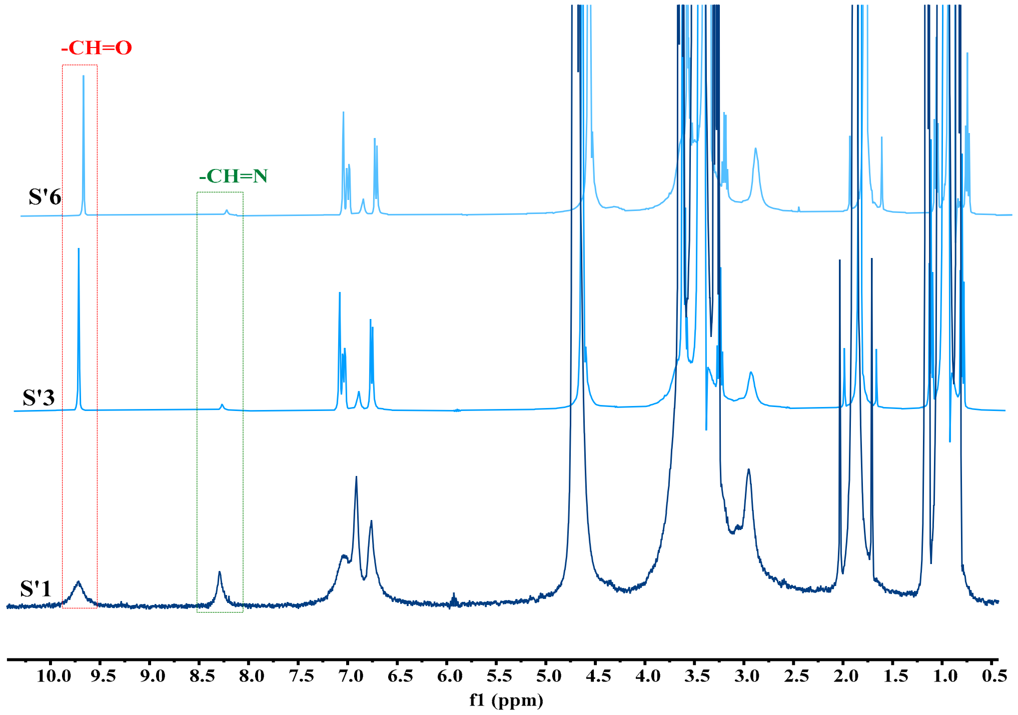

a)

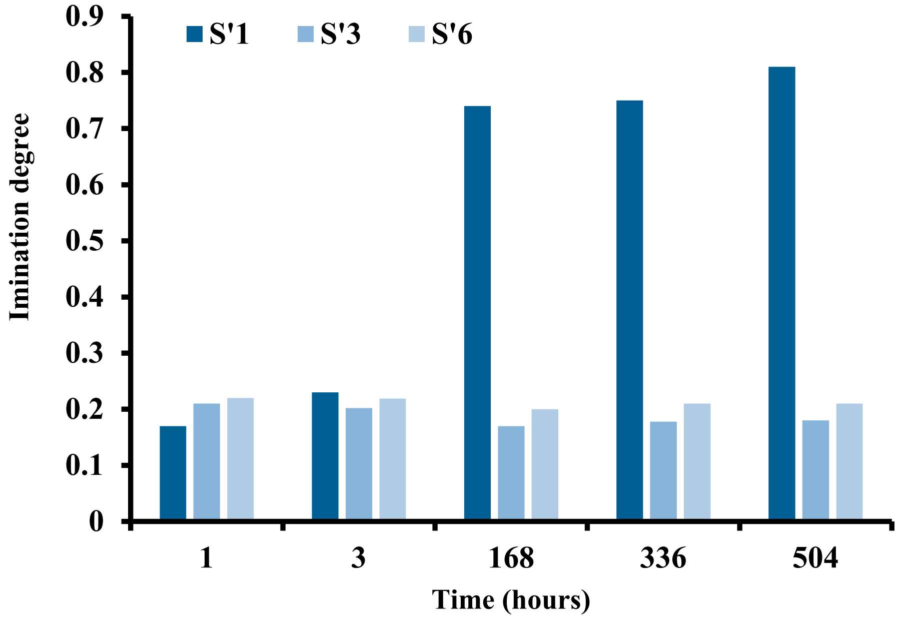

b)

Figure S6. a) ^{1}H-NMR spectra of the hydrogels S'$_x$ and b) Graphic representation of imine degree over time

The determination of chitosan's molecular weight *via* viscometric measurements

The molecular weight of chitosan was determined using a Schott CT 52 (Schott AVS 350) viscometer fitted with capillary No. 0. Different concentrations of chitosan (0.02, 0.04, 0.06, 0.08, and 0.1 g/dL) were prepared in a solution comprising 0.3 M acetic acid and 0.2 M sodium acetate and their viscosity was measured at 25°C. The intrinsic viscosity [η] was calculated from the graph of reduced viscosity *against* concentration (Figure S1).

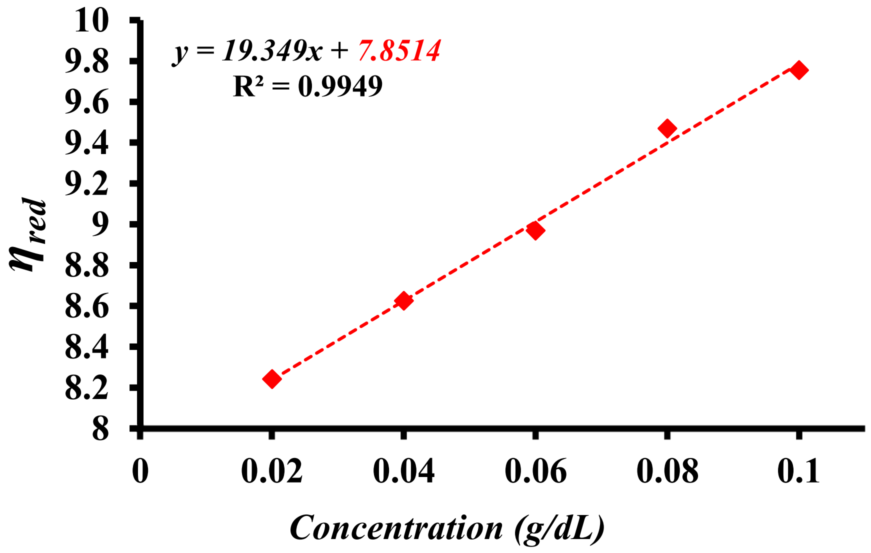

Figure S7. Graph of reduced viscosity *vs.* chitosan concentration

Utilizing the Mark-Houwink-Sakurada equation (Equation (S1)) and the empirically derived constants (k = 7.4 x 10^{-4} g/dL and a = 0.76), the molecular weight of chitosan was calculated based on the experimentally determined intrinsic viscosity (η = 7.8514) (Kasaai, 2007).

$$[\eta]=k\cdot[M_w]^{\alpha} \quad \textbf{\textit{(S1)}}$$

M_w= 198 kDa

The determination of the chitosan's deacetylation degree (DD)

To determine **DD**, 10 mg chitosan was dissolved in 1 mL of D_2O acidified with HCl (5µL). After stirring for 30 minutes, the solution was analysed using ^{1}H-NMR spectroscopy (Figure S2). The **DD** was calculated based on the relative intensities of peaks corresponding to acetylated and deacetylated units (Equation (S2)):

$$\boldsymbol{DD}\ (\%) = \left(1 - \frac{\frac{1}{3} \cdot I_{CH_3}}{\frac{1}{6} \cdot I_{(H_2 - H_6)}}\right) \cdot 100 \qquad \text{(S2)}$$

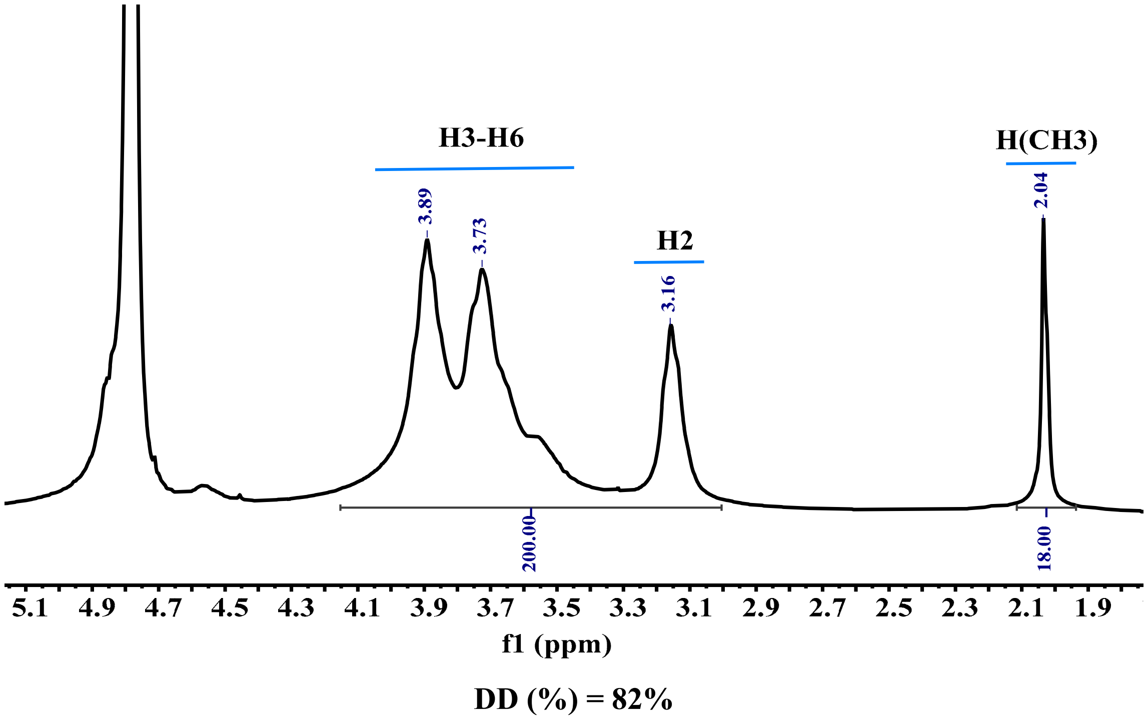

Figure S8. ^{1}H-NMR spectrum of chitosan

Model reaction of 5-methoxysalicylaldehyde (A) with ethanol

The possibility that the aldehyde would react with ethanol was investigated by NMR (Figure S9). The mixture was kept at 55°C for 3 h, following the same experimental protocol as in the case of the hydrogels. The NMR spectrum didn't present any chemical shifts besides the ones corresponding to the aldehyde and ethanol. Moreover, the integrals of the peaks indicate that the compounds do not react with each other and they form a physical mixture.

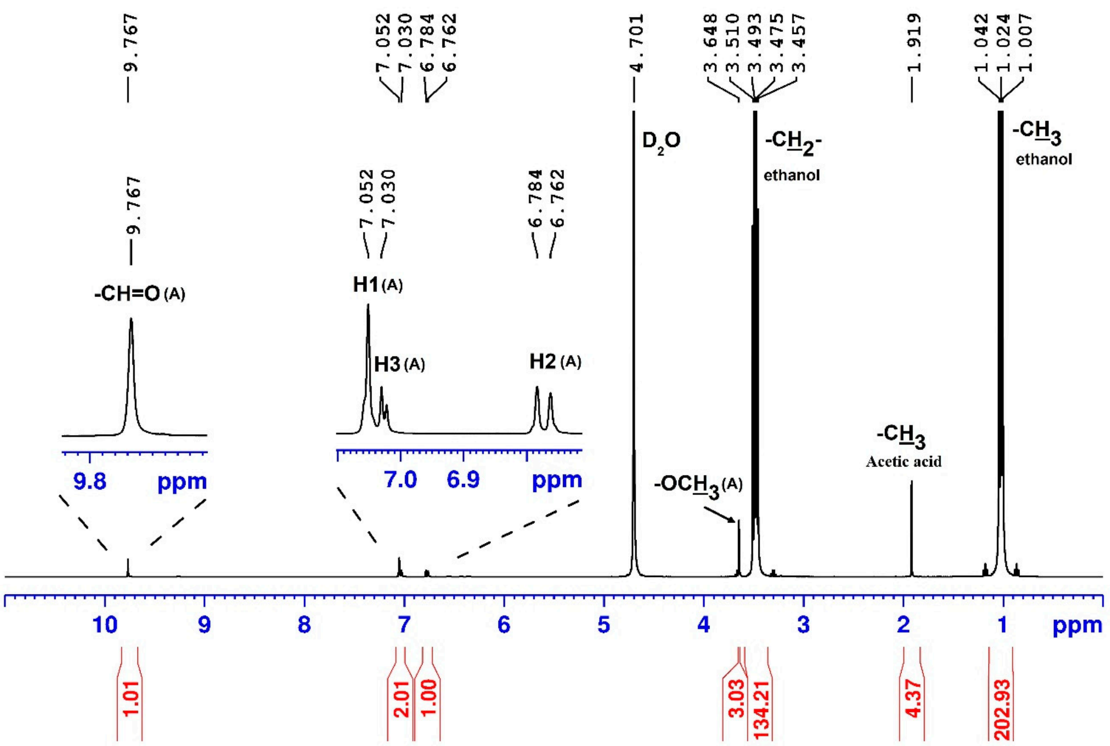

Figure S9. ^{1}H-NMR spectra of a mixture of aldehyde and ethanol in deuterium oxide, with traces of acetic acid.

Table S1. MES values of the xerogels in different media

MES / Samples	PBS (SD)	pH=5.5 (SD)	H_2O (SD)
S1	9 (±0.6)	6 (±0.7)	12 (±1.1)
S3	24 (±0.05)	31 (±1.4)	33 (±1.5)
S6	29 (±1.4)	51 (±1.1)	55 (±1.3)

Table S2. Cumulative aldehyde release (%) in different media

A% / Samples	PBS (SD)	pH=5.5 (SD)	H_2O (SD)
S1	9 (0.9)	15 (0.02)	4.5 (0.1)
S3	10 (0.9)	19 (0.44)	5 (0.4)
S6	14 (0.7)	41 (0.8)	9 (0.89)

Table S3. Parameters from fitting mathematical models to the *second stage* of aldehyde release in different pH media: a) H_2O, b) PBS (pH=7.4) and c) Acetate buffer (pH=5.5)

Model / Code	Zero Order		First Order		Higuchi		Korsmeyer-Peppas			Hixson-Crowell	
Second stage	R^2	K_0	R^2	K	R^2	K_H	R^2	K	n	R^2	K
**a) H_2O**											
S1	0.74	0.006	0.74	$-7x10^{-5}$	0.86	0.14	0.92	0.02	0.19	0.74	-0.00009
S3	0.71	0.003	0.71	$-2x10^{-5}$	0.84	0.07	0.93	0.04	0.08	0.71	-0.00005
S6	0.52	0.001	0.52	$-9x10^{-6}$	0.66	0.02	0.79	0.09	0.02	0.52	-0.00001
**b) PBS (pH=7.4)**											
S1	0.74	0.006	0.74	$-7x10^{-5}$	0.86	0.14	0.76	0.05	0.13	0.3	-0.00004
S3	0.53	0.007	0.53	$-1x10^{-4}$	0.66	0.17	0.51	0.07	0.05	0.53	-0.00001
S6	-	-	-	-	-	-	-	-	-	-	-
**c) Acetate buffer (pH=5.5)**											
S1	0.85	0.014	0.87	-0.0002	0.96	0.46	0.983	0.02	0.33	0.87	-0.0002
S3	0.85	0.01	0.86	-0.0001	0.96	0.33	0.985	0.07	0.15	0.86	-0.0002
S6	0.9	0.015	0.92	-0.0002	0.98	0.49	0.975	0.01	0.02	0.91	-0.0003
